# Supplementary material for: Wearable and Portable GPS Solutions for Monitoring Mobility in Dementia: A Systematic Review
Source: Sensors (Basel). 2022 Apr 27;22(9):3336. doi: 10.3390/s22093336 (PMC9104067; doi:10.3390/s22093336)
Supplement: Supplementary file 1 [file sensors-22-03336-s001.zip › sensors-1682187-supplementary.pdf]

Supplementary Materials: Search Terms

OVID Search Terms:

Databases: Ovid MEDLINE(R), AMED (Allied and Complementary Medicine), APA PsycInfo, Embase

1	Geographic Information System/
2	Global positioning system/
3	(GPS or gps or geographic Information System or Global Positioning system or differential Global Positioning system or global navigation satellite system or GLONASS or Geographic navigation satellite system or satellite communication system).mp.
4	((wearables or wearable* or wear* or portable or mobile or portab*) adj3 (sensors or sensor* or device or device* or electronic or electro*)).mp.
5	or/1-4
6	Dementia/
7	(dementi* or pseudodementia).mp.
8	Alzheimer's/
9	(alzheimer* or alzeimer* or (cortical adj4 sclerosis)).mp.
10	(((frontotemporal or (fronto adj temporal) or (corticobasal or (cortico adj basal) or (frontal adj lobe))) adj4 (degenerati* or dysfunction)) or ftld or ftlds or ftd or ftds).mp.
11	(huntington* or ((progressive or major or juvenile or hereditary) adj4 chorea)).mp.
12	("lewy bod*" or dlb or lbd or dlbd).mp.
13	(dementia adj3 (senile or presenile)).mp.
14	Vascular dementia/
15	Dementia, Vascular/
16	((dementi* or pseudodementia) adj3 vascular).mp.
17	((vascular or arteriosclerot* or multi-infarct* or multiinfarct* or multi infarct*) adj4 dementia*).mp.
18	("preclinical dementia" or "pre-clinical dementia").mp.
19	(prodrom* adj2 dement*).mp.
20	Mild Cognitive Impairment/
21	(cognitive impairment or cognitive decline).mp.
22	(cognitive* adj2 impair*).mp.
23	or/6-22
24	5 and 23
25	Animals/ not Humans/
26	24 not 25
27	General practice/
28	26 not 27
29	limit 28 to yr="2000 -Current"

iEEE Search Terms

(("All Metadata": Geographic Information System/ OR Global Positioning System/ OR GPS OR gps OR differential Global Positioning system OR global navigation satellite system OR GLONASS OR Geographic navigation satellite system OR satellite communication system OR wearables OR wearable* OR wear*)
AND
("All Metadata": Dementia/ OR Alzheimer's/ OR frontotemporal OR huntington OR huntington's OR "lewy bod*" OR Vascular dementia/ OR pseudodementia OR "preclinical dementia" OR "pre-clinical dementia" OR Mild Cognitive Impairment/ OR cognitive impairment OR cognitive decline))
NOT
(("All Metadata": Animals/ not Humans/)
OR
(General practice/))
